# Past, present, and future of Bcr-Abl inhibitors: from chemical development to clinical efficacy

**DOI:** 10.1186/s13045-018-0624-2

**Published:** 2018-06-20

**Authors:** Federico Rossari, Filippo Minutolo, Enrico Orciuolo

**Affiliations:** 1 Institute of Life Sciences, Scuola Superiore Sant’Anna, Piazza Martiri della Libertà, 33, 56127 Pisa, PI Italy; 2 University of Pisa, Pisa, Italy; 3 Department of Pharmacy, University of Pisa, Pisa, Italy; 4 Department of Clinical and Experimental Medicine, Section of Hematology, Azienda Ospedaliero Universitaria Pisana, Pisa, Italy

**Keywords:** Bcr-Abl, Structure-activity relationship, Leukemia, Targeted therapy, Tyrosine kinase inhibitors (TKIs), Imatinib, Dasatinib, Nilotinib, Bosutinib, Ponatinib

## Abstract

Bcr-Abl inhibitors paved the way of targeted therapy epoch. Imatinib was the first tyrosine kinase inhibitor to be discovered with high specificity for Bcr-Abl protein resulting from t(9, 22)-derived Philadelphia chromosome. Although the specific targeting of that oncoprotein, several Bcr-Abl-dependent and Bcr-Abl-independent mechanisms of resistance to imatinib arose after becoming first-line therapy in chronic myelogenous leukemia (CML) treatment.

Consequently, new specific drugs, namely dasatinib, nilotinib, bosutinib, and ponatinib, were rationally designed and approved for clinic to override resistances. Imatinib fine mechanisms of action had been elucidated to rationally develop those second- and third-generation inhibitors. Crystallographic and structure-activity relationship analysis, jointly to clinical data, were pivotal to shed light on this topic. More recently, preclinical evidence on bafetinib, rebastinib, tozasertib, danusertib, HG-7-85-01, GNF-2, and 1,3,4-thiadiazole derivatives lay promising foundations for better inhibitors to be approved for clinic in the near future.

Notably, structural mechanisms of action and drug design exemplified by Bcr-Abl inhibitors have broad relevance to both break through resistances in CML treatment and develop inhibitors against other kinases as targeted chemotherapeutics.

## Background

The vast majority of chronic myelogenous leukemia (CML) cases and 20–30% of those of acute lymphoblastic leukemia (ALL) are caused by a reciprocal chromosomal translocation between chromosome 9 and 22—t(9, 22)—thus forming the so-called Philadelphia chromosome (Ph) [1]. The product of this genetic rearrangement consists in Bcr-Abl fusion protein with deregulated tyrosine kinase activity that leads immune precursors to divide endlessly. That was the first innovative prove of a disease to be caused and marked by an acquired chromosomal translocation.

As the fusion protein was recognized to be the *primum movens* of those leukemias in the 1980s, the therapeutic effort was directed towards that specific target, trying to emulate the successful breakthrough of tamoxifen in breast cancer, the very first “targeted therapy” [2]. Imatinib (STI571) was therefore discovered as the first selective Bcr-Abl tyrosine kinase inhibitor (TKI) by means of drug screening approach [3, 4].

Despite the increase in overall survival allowed by imatinib [5], drug resistance onset led scientists to investigate imatinib fine structural mechanism of action to develop new and more effective compounds against mutated forms of Bcr-Abl. Most of resistances rely on Bcr-Abl aminoacidic substitutions, mainly within the kinase domain. One of the most frequent mutations, ranging from 2 to 20% of CML cases [6], is T315I (isoleucine replaces threonine in position 315 of Bcr-Abl), which is also the deadliest case since it leads to resistance to second-generation TKIs, such as nilotinib and dasatinib [7, 8]. Only with the advent of ponatinib has it been possible to overcome that further therapeutic hurdle [9]. Therefore, Bcr-Abl inhibitors represent a model for paving the way towards the development of new small molecules for targeted therapy.

Here, we review the rational development of the latter TKIs that allow the already-high CML survival to become even higher, approaching totality of cases [10]. Specific in vitro potency of TKIs will be compared in term of IC_50_ in cell proliferation assays testing target kinases (50% inhibitory concentration (IC_50_)is defined as the drug concentration resulting in 50% cell growth inhibition that corresponds to the fraction affected of 0.5). IC_50_ values of the debated TKIs are summarized in Table 1. Clinical effects will instead be reported accordingly to the end points of the most authoritative trials on the subject.

**Table 1:** Activity of tyrosine kinase inhibitors against wild-type and mutated kinases, expressed as IC_50_ (nM) in cellular assays

	Kinase	TKI											
		Imatinib	Nilotinib	Dasatinib	Bosutinib	Ponatinib	Bafetinib	Rebastinib	Tozasertib	Danusertib	HG-7-85-01	GNF-2 + dasatinib (dasatinib concentration)	GNF-5 + nilotinib (nilotinib concentration)
	Abl												
	WT	100–500	< 10–25	0.8–1.8	41.6	0.5	72	19–80	10–104	26–360	58.5	100 (2 nM)	30 (1 μM)
P-loop	M244V	1600–3100	38–39	1.3	147.4	2.2	240	78–90					
	L248V	1866–10,000	49.5–919	9.4	145.6	1.7							
	G250E	1350–> 20,000	48–219	1.8–8.1	179.2	4.1	160	98–600	180				
	Q252H	734–3120	16–70	3.4–5.6	33.7	2.2	410	24–190	130		50–100		
	Y253F	> 6400–8953	182–725	6.3–11	40	2.8	81	39	80				
	Y253H	> 6400–17,700	450–1300	1.3–10	24.9	6.2		56–300	190		500–1000		
	E255K	3174–12,100	118–566	5.6–13	394	14	540	127–251	84	470	500–1000		
	E255V	6111–8953	430–725	6.3–11	230.1	36	1400	850					
C-helix	D276G	1147	35.3	2.6	25	1.05							
	E279K	1872	36.5–75	3	39.7	1.5							
ATP-binding region	V299L	540–814	23.7	15.8–18	1086	0.3		72	200		500–1000		
	F311L	480–1300	23	1.3				140					
	T315I	> 6400–> 20,000	697–> 10,000	137–> 1000	1890	11	> 10,000	13–200	30–74	120	140	3300 (1 μM)	300 (1 μM)
	T315A	125	27–67.5	760	249.6	1.6		19–64	88				
	F317L	810–7500	39.2–91	7.4–18	100.7	1.1	760	36–280	84		500–1000		
	F317V	500	350	17–38	478.4	10		223					
Catalytic segment	M351T	880–4900	7.8–38	1.1–1.6	29.1	1.5	150	14–86	65	510	250–500		
	F359V	1400–1825	91–175	2.2–2.7	38.6	10	1300	138–350					
	V379I	1000–1630	51	0.8									
Activation loop	L384M	674–2800	39–41.2	4	19.5	1.1							
	L387M	1000–1100	49	2									
	H396R	1750–5400	41–55	1.3–3	33.7	2.95		290					
	H396P	850–4300	41–43	0.6–2	18.1	1.1	95	66–81	160				
C-term	F486S	2728–9100	32.8–87	5.6	96.1	1.05	470				500		
	cKit (CD117)												
	WT	100–150	14.7	79	6313	12.5	840	424–538			> 1000		
	D816V	3800	500	37	2772	72–143	3800		100				
	V560G	75	108	585	181	165	51		> 2000				
	V559D	3927	297	432		11					250–500		
	PDGFR α												
	WT	100	3–71	13–16	> 10,000	1.1	56	60–80					
	T674I	> 5000	376	> 500		9					6.25		
	D842V	642	1310	62		154	1281				1000		
	V561D	10	10				59				> 1000		
	PDGFR β												
	WT	39	60.11	4	> 10,000		> 1000	103–123			< 100		
	T681I	> 25,000									> 1000		
	Aurora A, B, C							> 5000	4–27	13–79			
	Src	> 10,000		0.8	1.2	2.2	1700	34					
	Lyn	352	1281				19	29					
References		[20, 69–72]	[20, 69, 71, 73–75]	[69, 76–78]	[69, 79, 80]	[69, 79, 81, 82]	[36, 70, 83]	[39, 40, 84]	[40, 85]	[48, 86]	[50]	[53]	[87]

Here, IC_50_ values related to cell growth assays of tyrosine kinase inhibitors (TKIs) against their main targets, both unmutated (WT) and mutated, are shown in nanomolar units. Regarding Abl kinase, domains harboring specific mutations are displayed on the left of the table. Range values represent either intra- or inter-study variability, while the missing ones are still unavailable to the best of our knowledge, representing putative objectives to be determined in future studies. The activity spectrum of the newest inhibitors, namely rebastinib, tozasertib, danusertib, HG-7-85-01, and GNF, should be characterized in further details to fill the current data gap

## Main text

### Structural data of Bcr-Abl fusion protein

**Fig. 1:**
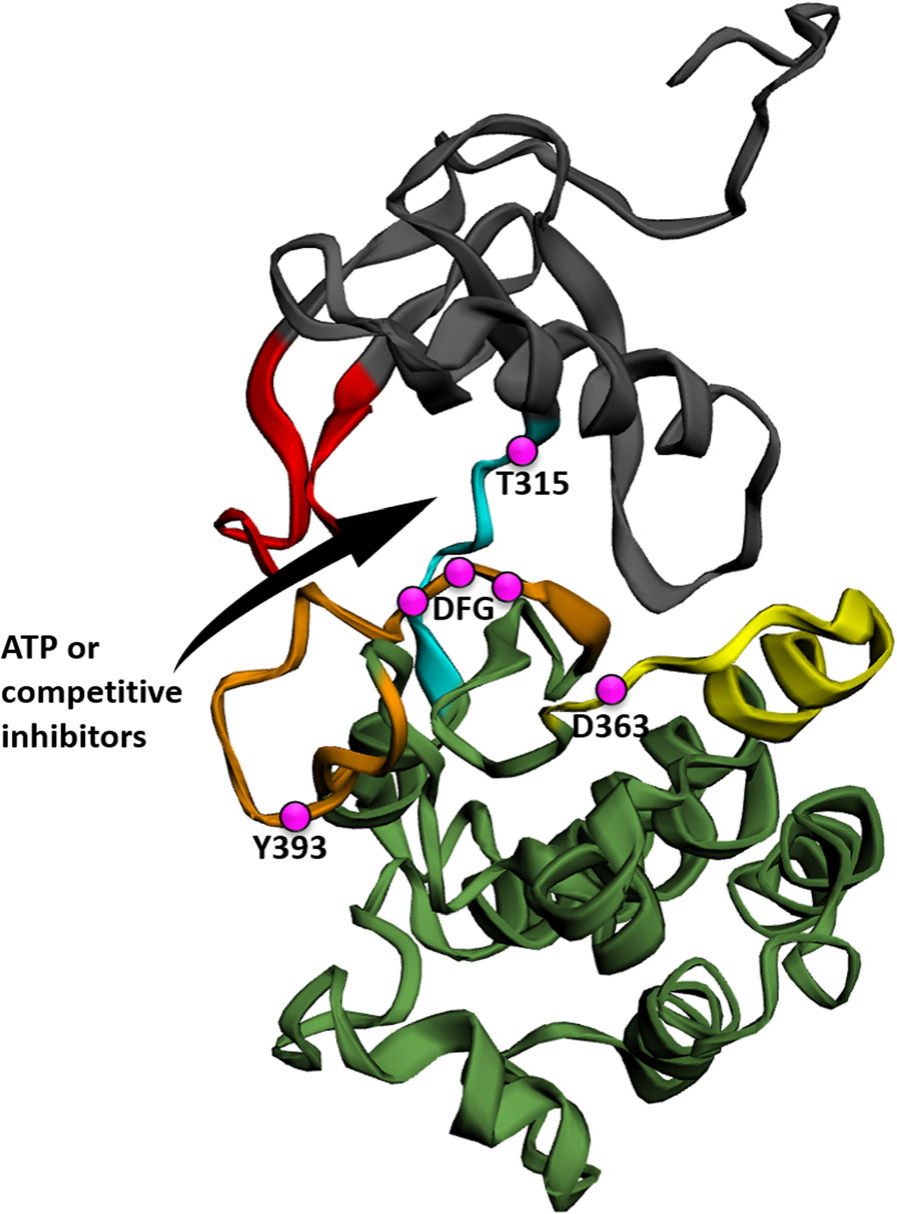
Structural 3D model of Bcr-Abl catalytic domain. The ribbon diagram of crystal structure shows the *N-*lobe at the top (dark gray) and *C-*lobe at the bottom (green), rich in β-sheets and α-helices respectively. The catalytic segment (yellow), the P-loop (red), the activation loop (orange), and the hinge region (light blue) stand in the middle. Key amino acidic residues are indicated in magenta circles: Thr315 (T315) is the gatekeeper residue within the ATP-binding pocket (black arrow), Asp363 (D363) is pivotal for nucleophilic attack on peptide substrate during catalysis, Tyr393 (Y393) is the target of phosphorylation that controls Abl activation and inactivation, whereas the DFG (Asp-Phe-Gly) motif coordinates fundamental cofactors for catalysis, namely Mg^2+^ ions [88]

Crystallographic analysis of Bcr-Abl protein highlights a two-lobe catalytic domain: *N-* and *C-*lobes towards *N-* and *C-*terminus of the sequence, respectively (Fig. 1). β-Sheets compose the former, whereas α-helices prevail in the latter. An important Wolker loop (also known as phosphate-binding or P-loop) links two β-sheets of *N-*lobe. Thanks to its high flexibility, a P-loop residue can interpose between β- and γ-phosphates of bound adenosine triphosphate (ATP), thus promoting phosphoric anhydride bond break after nucleophilic attack from Asp363 of the so-called catalytic loop [11]. The hinge region, which links the two lobes, also participates in ATP binding by two hydrogen bonds. Within the ATP-binding pocket, a “gatekeeper” residue, Thr315, interacts with ATP, too; furthermore, it plays a key role in conferring selectivity to some of the Bcr-Abl inhibitors. Indeed, Thr315 is located at the peak of one of the multiple hydrophobic “spikes” connecting *C-* and *N-*lobes in active conformation [12].

At one portion of the *C-*lobe, a pivotal loop with regulatory function stems out. Thanks to its mobility, this “activation loop” can alter its conformation to activate and inactivate the kinase. On a structural point of view, the activation loop has in turn three key portions: a DFG (Asp-Phe-Gly) motif at the *N-*terminus, a central tyrosine residue (Tyr393), and the peptide substrate-binding *C-*terminus [11]. During active phase, the former contributes with its Asp381 residue in coordinating Mg^2+^ ions, key cofactors of catalysis, whereas the latter accommodates the peptide substrate to be phosphorylated. Tyr393, instead, is the Abl target residue whose phosphorylation leads to loop extension and assumption of kinase active conformation. This conformation shows a high grade of similarity among various kinase families, thus justifying the greater number of TKIs developed towards the conversely more characteristic inactive conformation. In fact, the latter conformation displays the activation loop folding in towards ATP-binding site, hence avoiding ATP entrance. This arrangement dislocates the DFG motif out of the catalytic site (here the name “DFG-out” conformation) and prevents Mg^2+^-mediated catalysis. Conversely, the active conformation is also known as “DFG-in,” since the activation loop protrudes out from the ATP-binding pocket, confining DFG motif inside the catalytic site. While the latter conformation is shared by different kinases, the former defines a peculiar site among the dislocated activation loop, the gatekeeper residue, and the *C-*lobe, which has been set as the main target in the development of specific TKIs [13].

**Fig. 2:**
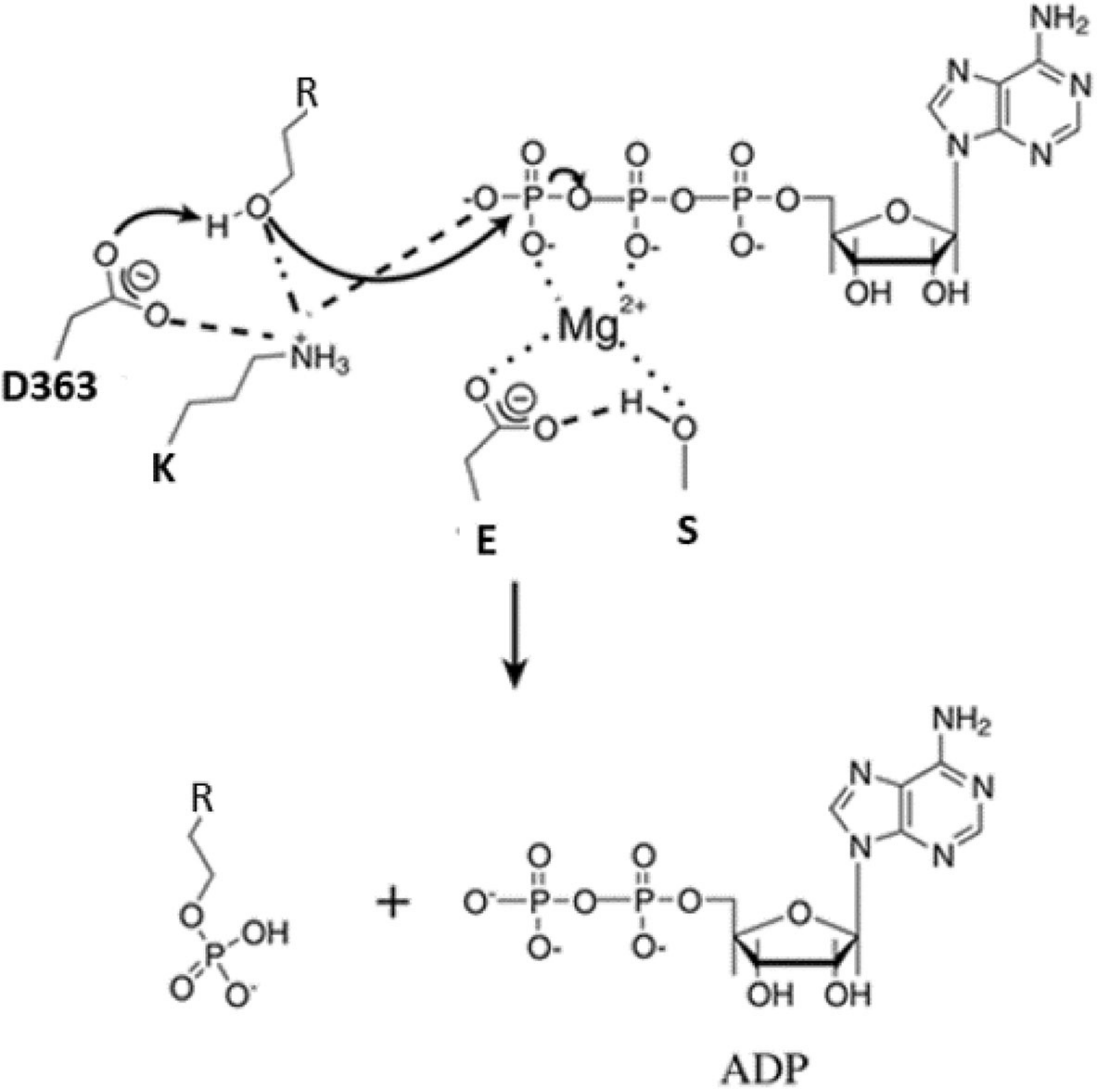
Asp363 protonation during catalysis. Here, the reaction mechanism of substrate phosphorylation is shown. The nucleophilic attack of D363 on hydroxyl group of peptide substrate leads to its transient protonation that in turn causes conformational changes to inactive state. (R = peptide substrate, D = aspartate, K = lysine, E = glutamate, S = serine)

Counterintuitively, even if the chimeric oncoprotein is known to be hyperactive to cause leukemia, after each substrate phosphorylation step, the Asp363 residue gets transiently protonated (Fig. 2), leading to conformational changes and a consequent inactivation, which allows inhibitor binding. This represents the reason why the DFG-out inhibitors are effective despite the kinase hyperactivity in the tumor. As previously told, the Thr315 residue is pivotal in stabilizing active conformation: its replacement by isoleucine (T315I) prevents conformational changes to inactive form, therefore conferring resistance to several DFG-out inhibitors [12].

### First-generation inhibitor: imatinib

**Fig. 3:**
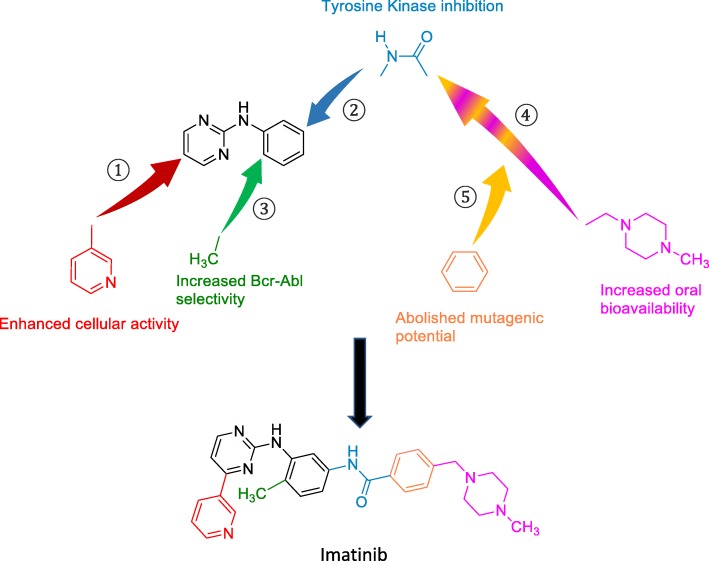
Chemical optimization and functions of imatinib structure. The phenylaminopyrimidine derivative lead compound is indicated in black. ① The pyridyl group (red) added at 3′-position of the pyrimidine moiety enhanced cellular activity, ② the amide substituent (blue) on the phenyl ring provided the molecule with inhibitory activity against tyrosine kinases, and ③ the 6-methyl (green) addition to the central aminophenyl ring nullified the unspecific activity on PKC, thus increasing selectivity of the compound for Bcr-Abl. Finally, ④ an *N*-methylpiperazine (purple) was added to enhance aqueous solubility and oral bioavailability of the drug, but ⑤ required the insertion of the amide linker and a benzene ring (yellow) as a spacer to abolish the mutagenic potential of the aniline moiety otherwise obtained. Imatinib was therefore developed as optimized Bcr-Abl oral inhibitor

In the early 1990s, a screening for protein kinase C (PKC) inhibitors was carried out and led to the identification of a phenylaminopyrimidine derivative as potential lead compound with high prospective for diversity, allowing simple chemistry to produce more potent and selective molecules against several kinases [14]. At first, a pyridyl group was added at the 3′-position of the pyrimidine to boost its cellular activity. Various functional groups were then tested as substituents in the phenyl ring, until the presence of an amide group was found to confer inhibitory action against tyrosine kinases. Furthermore, analysis of structure-activity relationships evidenced that a substitution in position 6 of the diaminophenyl ring abolished the activity against PKC. Conversely, the addition of a methyl group in an *ortho* position to the amino group increased selectivity for Bcr-Abl. However, the resulting molecule still showed poor oral bioavailability and solubility in water, which were considerably improved by the introduction of an *N*-methylpiperazine group. Nevertheless, in spite of the abovementioned improvements and of the increased affinity of the resulting molecule for its target, the *N-*methlypiperazine addition would have generated an aniline moiety in the structure. To abolish its mutagenic potential, the abovementioned amide group and a spacer benzene ring were introduced [14]. These structural developments led to the production of imatinib (STI571) (Fig. 3), the first known ATP competitor able to inhibit Bcr-Abl kinase with high selectivity (but not absolute specificity: wild-type (WT) platelet-derived growth factor receptor (PDGFR) and c-Kit are inhibited, too, as demonstrated by similar IC_50_ values of approximately 100–150 nM for all three kinases [15], see Table 1) and to be approved in the clinics in 2001, less than a decade later its experimental production [5, 16]. Docking studies and X-ray crystallography evidence that imatinib interacts with its target through binding the hinge region in its entire width [17].

Imatinib consists in a typical bisarylanilino core comprising a phenyl ring on one side and a pyridine-pyrimidine moiety on the other side, possessing a benzamide-piperazine group in the meta-position of the aniline-type nitrogen atom (Fig. 3). The core interacts with gatekeeper Thr315 through both hydrogen bond (H-b) and Van der Waals (VdW) interactions. The two substituents, instead, are splayed at about 120°, fitting the adenine-binding site and the abovementioned peculiar site of the DFG-out conformation, respectively. The former is shielded from the solvent in a hydrophobic cage delimited by Tyr253 of the P-loop (which is kinked during the inactive phase), Phe382 of the activation loop, together with Leu248, Phe317 and Leu370 residues; the latter preeminently establishes VdW interactions with the following Bcr-Abl residues: Val289 and Met290 of the *C-*lobe, Asp381 of the DFG motif, and Ile360 and His361 of the catalytic loop [11].

Overall, the majority of imatinib interactions are of weak VdW type, but also six highly energetic H-bs take place: each of these accounts for a relative high portion of the total binding energy of the complex kinase-inhibitor, thus providing a theoretical explanation for resistance due to mutations of H-b donor/acceptor residues. In fact, if one of these bonds and consequently its energetic contribution gets lost, free energy of dissociated state becomes highly competitive against that of the bound state, thus justifying the missed interaction between imatinib and the mutated kinase due to less favorable thermodynamic factors: that underlies resistance.

### Mechanisms of resistance to imatinib

Imatinib treatment fails in approximately one third of patients [18]. Underlying mechanisms of resistance are classically divided into two types: Bcr-Abl-dependent and Bcr-Abl-independent mechanisms. The latter consists mainly in increased drug efflux/decreased uptake and activation of alternative onco-pathways. The former, instead, are mainly due to point mutations of Bcr-Abl that alter inhibitor binding or conformational changes; nevertheless, a residual amount of Bcr-Abl-dependent resistances are also due to gene amplification or hyperexpression [19].

Since the vast majority of cases are due to point mutations, new inhibitors have been developed with a rational drug design approach aimed at overriding resistances by loosening conformational and binding requirements without losing specificity. Second-generation inhibitors solve almost the entirety of mutations except for T315I. The substitution of the gatekeeper residue frustrates the action of inhibitors through two potent mechanisms: break of a H-b and strong stabilization of the active DFG-in conformation. This consistent obstacle has been overcome only thanks to third-generation inhibitors [20].

### Clinically approved second-generation inhibitors

In order to break through mutations, several second-generation TKIs have been developed and approved for clinics, i.e., nilotinib and dasatinib as either first or second line of treatment, and bosutinib as second line only [21].

**Fig. 4:**
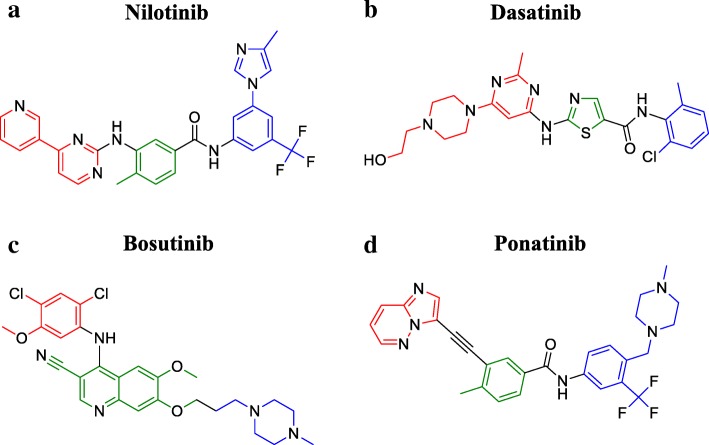
**a**–**d** Structure comparison of Bcr-Abl clinically approved inhibitors. Chemical structures are here represented in color code with regard to analogous groups of different tyrosine kinase inhibitors (green: core structure; red and blue: substituents group)

Nilotinib (AMN107) shows greater potency and effectiveness against almost the totality of resistance-conferring mutations (see Table 1 for IC_50_ values), except for T315I and few others, in newly diagnosed Ph+ CML patients [22, 23]. This result has been reached starting from the structure of imatinib by inverting the amide linking group, by replacing the piperazine ring with 3-methylimidazole, and by adding trifluoro-methyl group to the anilinocarbonyl substituent, in order to increase the number of VdW interactions (Fig. 4a). Therefore, energetic contribution to the total of each H-b decreases, avoiding impairment of inhibitor binding in case of mutation of key residues involved in H-b interactions, although the overall number of H-bs was kept unchanged. In spite of these modifications, less stringent binding requirements did not compromise selectivity and potency of nilotinib, which conversely are even increased when compared to those of imatinib (IC_50_ values of 10–25 and 100–500 nM, respectively—see Table 1). As previously told, nilotinib is active against DFG-out conformation only, and this accounts for T315I resistance. Interestingly, nilotinib is not substrate of neither influx transporter nor efflux P-glycoprotein pump, unlike imatinib and, therefore, is not sensitive to Bcr-Abl-independent mechanisms of resistance [24].

Dasatinib (BMS-354825) is a peculiar DFG-in inhibitor, even though it is not effective in the case of T315I mutation [22]. Compared to imatinib, dasatinib enables patients with chronic phase CML to achieve faster and deeper treatment responses (i.e., median time to major molecular response (MMR) for dasatinib arm was 15 versus 36 months for the imatinib arm, and BCR-ABL transcript 4.5-log reduction was achieved by 17 versus 8% of patients by 24 months, respectively) [25]. Structurally, the core phenyl ring has been replaced by an aminothiazole group which occupies the adenine pocket of Abl (Fig. 4b). The pyridine group of imatinib is instead replaced by a hydroxyethyl piperazine, which remains solvent-exposed also after Bcr-Abl binding. Dasatinib is a smaller molecule than imatinib and it establishes less interactions with its targets: nuclear magnetic resonance studies have evidenced that dasatinib binds Bcr-Abl very versatilely, in both active and inactive conformations [26]. However, the free inactive DFG-out conformation has a higher entropy than the active conformation: the drop in entropy is less pronounced after dasatinib binding if the target is active, with enthalpy variation being very similar in both conformations. Thus, free energy decreases more in case of binding during DFG-in phase, which is therefore preferentially inhibited by dasatinib, because it is thermodynamically favored. Consequently, several conformation-altering mutations, except for T315I, are susceptible to dasatinib action, anyway [27].

Bosutinib (SKI-606) has a more different structure (Fig. 4c), since it has been developed from a leading Src inhibitor compound (4-[(2,4-dichlorophenyl)amino]-6,7-dimethoxy-3-quinolinecarbonitrile) [28]. The quinoline central core required the addition of a hydrophilic protonable *N*-methylpiperazino moiety. Though it is not effective against T315I mutation and does not show total selectivity for Bcr-Abl (see Table 1), it has the important advantage of being not efficiently excreted by multidrug resistance transporters [29, 30]. Bosutinib is currently approved as second-line treatment of CML [21]. Recent data suggest it can be an important alternative to imatinib for previously untreated patients with chronic phase CML, given its earlier and higher rate of responses (47.2 and 36.9% of MMR at 12 months for bosutinib and imatinib, respectively) [31].

### Third-generation inhibitor: ponatinib

To date, the only approved third-generation TKI is ponatinib (AP24534), a dual Src/Abl inhibitor designed to especially overcome T315I mutation. In fact, isoleucine in position 315 complicates Bcr-Abl switching to inactive conformation and H-b formation with DFG-out inhibitors. Nonetheless, ethynyl linkage of ponatinib has indeed been inserted to accommodate isoleucine side chain without any steric interference also in inactive conformation (DFG-out) [32]. Structurally (Fig. 4d), it nicely overlaps to nilotinib with only small differences other than ethynyl linker: the methyl imidazole group is replaced by a methyl piperazine moiety (like in imatinib). Besides, instead of the pyridine-pyrimidine group of nilotinib, ponatinib has a terminal imidazo[1,2-b]pyridazin portion in the same position to optimize H-b formation within the hydrophilic pocket in which it gets accommodated. Other bonds are similar to those of nilotinib and so abundant that kinase point mutations have less effect on the overall binding affinity and the potency of the drug (see Table 1, ponatinib IC_50_ are the lowest for almost every Bcr-Abl point mutation) [33]. Clinically, all these structural modifications result in a high activity in heavily pretreated patients with Ph+ leukemias with resistance to other inhibitors, including patients with T315I mutation, other mutations, or no mutations (in [34], out of 43 patients with those characteristics, 98% had a complete hematologic response, 72% a major cytogenetic response, and 44% a MMR). Moreover, ponatinib has recently been demonstrated a valuable alternative to allogeneic stem cell transplantation in patients with T315I-positive advanced CML and Ph+ ALL [9].

### Preclinically validated inhibitors: the targeted therapies of tomorrow?

Other molecules have been effectively tested since the advent of second-generation inhibitors, but have not entered common clinical practice for neither CML nor ALL, yet. Namely, they are bafetinib, rebastinib, tozasertib, danusertib, HG-7-85-01, GNF-2 and -5, and 1,3,4 thiadiazole derivatives. Furthermore, it is highly probable that new in silico and in vitro evidences may lead to new molecules to enter the clinics in the near future to overcome persistent resistances.

**Fig. 5:**
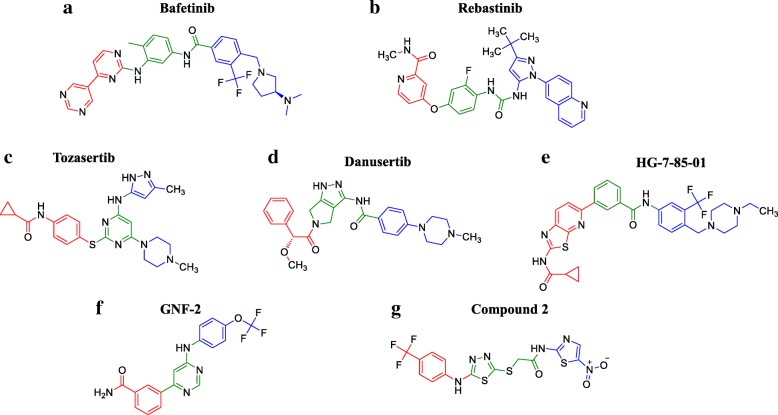
**a**–**g** Structure comparison of Bcr-Abl preclinically validated inhibitors. Chemical structures are here represented in color code with regard to analogous groups of different tyrosine kinase inhibitors (green: core structure; red and blue: substituents group)

Bafetinib (INNO-406) development was aimed at extending the susceptibility spectrum of mutations to TKIs and increasing selectivity towards Bcr-Abl to reduce clinical adverse reactions during treatment, e.g., cardiovascular and metabolic toxicities of nilotinib [35]. That was pursued by increasing hydrophobic properties of the benzamide ring of imatinib (Fig. 5a): a trifluoro-methyl group, similarly to nilotinib, was added to increase VdW interactions in the abovementioned “hydrophobic cage” [36]. In the meantime, in light of X-ray crystallography predictions, the pyridine group of imatinib was replaced by a more hydrophilic pyrimidine ring, thus increasing aqueous solubility without impairing binding properties and potency against Bcr-Abl (IC_50_ 71 nM) [17]. Finally, the dimethylaminopyrrolidine portion took the place of the N-methylpiperazine ring, favoring H-b formation [36]. In this way, its activity is retained against several Bcr-Abl mutants (submicromolar IC_50_, see Table 1), with the exception of a minority including T315I (IC_50_ > 10 μM) [37]. A phase I clinical trial evidenced that 19% of CML and Ph+ ALL patients with imatinib resistance or intolerance reached the complete cytogenetic response after bafetinib as second-line treatment, suggesting its potential clinical efficacy [38]. Differently from some solid tumors, a phase II trial for CML and ALL is not ongoing, yet.

Rebastinib (DCC-2036) is a non-competitive conformational control inhibitor, designed to overcome Abl gatekeeper mutations, mainly T315I, that impede the occurrence of the DFG-out conformation and the inhibitory action of both first- and second-generation inhibitors [39]. It stabilizes a fundamental bond for inactive conformation between Glu282 and Arg386, regardless gatekeeper mutations. Structurally, the fluoro-substituted phenyl central core, possessing a ureic linker in *ortho* to the halogen atom, is also bound to a carboxamide-substituted pyridine on one side, and to a pyrazole, bearing 4-*tert*-butyl and 1-(6-quinolinyl) substituents, on the other side (Fig. 5b). Crystallography evidenced that the ureic and the carboxamide-pyridine groups establish five H-bs mainly with the aforementioned Glu282 and Arg386 residues, whereas the rest of the molecule optimizes VdW interactions with a hydrophobic cluster of amino acids, forcing out the DFG motif from the catalytic site. In the case of T315I mutation, the hydrophobic interactions are even enhanced, justifying sensitivity to rebastinib in cellular assays with clones displaying this mutation (IC_50_ 13 versus 19 nM for unmutated Abl) [40]. However, it is much less active against P-loop E255V mutation (IC_50_ 800 nM), possibly due to destabilization of Bcr-Abl inactive conformation, but its molecular mechanism deserves further characterization [39]. Clinically, although rebastinib showed efficacy (of 40 CML patients, 8 complete hematologic responses were achieved, 4 of which had a T315I mutation) [41], benefit has been considered insufficient to justify continued development against leukemias since the advent of ponatinib, to date.

Tozasertib (MK-0457, VX-680) is a pan-Aurora Kinase inhibitor (IC_50_ 4–27 nM) with activity also on Abl (IC_50_ 10 nM) as ATP competitor [37, 42]. Peculiarly, Aurora kinases are inhibited in their inactive state, whereas Bcr-Abl, both WT and mutant T315I, in active conformation [37]. Indeed, co-crystal structures evidenced four H-bs established by the aminopyrazole pyrimidine inhibitor (Fig. 5c) with key residues of the ATP-binding pocket, including Asp381 of the DFG motif when folded in the ATP site. Neither bonds nor steric hindrance occur between tozasertib and the gatekeeper residue, accounting for vulnerability of both WT and T315I Bcr-Abl (with IC_50_ of 30 nM) to this TKI [43]. All that led to a phase II clinical trial evidencing benefit for patients with T315I Bcr-Abl CML (44% had hematologic responses), suggesting even more efficacy than ponatinib for accelerated phase disease or as bridge therapy for stem cell transplantation, given its higher myelosuppressive effect [44]. Further developments or combinational regimens seem to be amply justified for this promising compound.

Danusertib (PHA-739358) is a multikinase inhibitor with a selective spectrum extended to Aurora kinases, Ret, TrkA, FGFR1, and Abl (IC_50_ of 13–79, 25, 31, 31, and 47 nM, respectively) [45, 46]. Similar to dasatinib and tozasertib, it is an ATP competitor for the active form of Bcr-Abl, as evidenced by crystallographic data [47]. Its pyrrolopyrazole core (Fig. 5d) provides three H-bs with the hinge region of Abl, whereas the benzyl group packs against Leu370 and the *N-*methyl-piperazine sticks out the kinase pocket to be solvated. No key interactions are established with the gatekeeper residue. Therefore, danusertib binding mode accommodates the T315I mutation, avoiding the steric clash that oppositely occurs between first-/second-generation inhibitors and the isoleucine 315 side chain. Actually, binding affinity for T315I mutant is even higher than that for WT Bcr-Abl (Kd 200 nM versus 2 μM, respectively [47]), possibly due to increase in VdW interactions between the inhibitor and the isoleucine residue. That accounts also for the higher potency shown in cellular assays against T315I mutant (IC_50_ 120 vs 360 nM of WT Abl [48]). Clinically, danusertib has shown acceptable dose-dependent toxicities and promising activity in advanced and resistant ALL and CML patients within a phase II trial (4 out of 29 patients with accelerated or blast phase CML responded, all 4 with T315I Bcr-Abl) [49], paving the way for further preclinical and clinical advancements.

HG-7-85-01 is a hybrid compound, designed by superposition of nilotinib and dasatinib structures. The master concept of condensing in a unique molecule the advantages of a combinational therapy, i.e., overcoming resistances, led to its first design and synthesis [50]. Structurally, the aminothiazole moiety of dasatinib is condensed to a pyridine ring and the resulting portion is linked to the phenyl-benzamide group of nilotinib (Fig. 5e), conferring selectivity for DFG-out conformation of Bcr-Abl. In fact, this structural arrangement results in a very accommodating inhibitor molecule for the gatekeeper residue, showing activity also against T315I Bcr-Abl in cellular assays (IC_50_ 140 nM, less than threefold higher than for WT, i.e., IC_50_ 58.5 nM [50]). Furthermore, the target selectivity spectrum is narrower than that of ponatinib at a high-throughput screening [50, 51], suggesting less adverse reactions than ponatinib at a clinical level, especially those related to cardiovascular toxicity. However, no clinical data are currently available, nor are there ongoing trials, to the best of our knowledge.

Recently, new state-of-the-art molecules have been designed and tested at a preclinical level to sensitize T315I mutation to first and second-generation TKIs. Genetic, nuclear magnetic resonance, crystallography, mutagenesis and mass spectrometry studies identified GNF-2 (Fig. 5f) and GNF-5 (its analogue bearing a *N*-(2-hyrdroxyethyl) group at the amidic nitrogen atom) to be two allosteric interactors of Bcr-Abl [52]. They bind the myristylation site of *C-*lobe inducing a forced conformational change of the kinase to inactive state, even if its gatekeeper residue is mutated [53]. Co-administration of a classical inhibitor with these compounds may therefore be effective also against T315I mutant. However, mutations around the myristate binding site, e.g., C464Y, P465S and V506L, are known to induce resistance to GNF-2/5 activity, putatively by steric hindrance (IC_50_ > 10 μM) [52]. This raises critical issues on some compound mutations of Bcr-Abl, i.e., the coexistence of different Bcr-Abl mutants in leukemic clones of a same patient, which necessarily need to be targeted by other approaches. Particularly, GNF-2 + dasatinib (IC_50_ = 100 nM with 2 nM dasatinib concentration) and GNF-5 + nilotinib (IC_50_ = 30 nM with 1 μM nilotinib concentration) resulted the most active and synergistic in vitro combinations [52, 53]. Clinical translation of these promising results could be found to be effective, especially for Ph+ ALL patients, since they often present the p190 Bcr-Abl variant on which these compounds seem to be more active.

A further way to inhibit Bcr-Abl could be paved by 1,3,4-thiadiazole derivatives, such as compound 2 (Fig. 5g), which emerged as putative DFG-in inhibitors from an in silico screening by molecular docking simulation studies [54]. Indeed, flexibility of its core is supposed to allow several conformations of the substances to bind the ATP site of Bcr-Abl active state [55]. As a matter of fact, compound 2 proved to be a better Bruton’s tyrosine kinase-inhibitor than imatinib (IC_50_ 1 vs 7 μM), but predictions about affinity while testing different substituents bound to the central thiadiazole core may lead to the development of even more effective Bcr-Abl inhibitors too, putatively also in the case of resistance to classical TKIs [54].

## Conclusions

Treatment of Ph+ CML and ALL has dramatically changed since the advent of targeted therapy against the Bcr-Abl fusion protein at the turn of the twenty-first century, and so the prognosis has done. The development of targeted inhibitors started from a high-throughput screening to find out a leading pharmacophore that includes compounds able to bind and block the chimeric kinase, by impeding ATP binding in a competitive manner [16, 17]. After its rational development, according to structure-activity relationship analysis and enzymatic assays [16], imatinib was optimized and rapidly approved for the clinic [5]. Actually, the IRIS trial clearly demonstrated that imatinib presented much higher effectiveness and reduced toxicity if compared to the standard of care of that time, i.e., IFNα plus low-dose cytarabine regimen [56].

Although the radical increase in mean survival, new mutations and forms of resistance came upon in common clinical practice [57], requiring further development of inhibitors, similar to the process that led from the parent compound to imatinib. By means of imatinib modification or exploitation of totally different molecular scaffolds, several second- and third-generation TKIs were developed. Some have already been approved for clinic use, i.e., the second-generation nilotinib, dasatinib, and bosutinib, and the third-generation ponatinib, whereas other still need clinical validation, e.g., bafetinib, rebastinib, tozasertib, danusertib, HG-7-85-01, GNF-2 and GNF-5, and other 1,3,4-thiadiazole derivatives. The onset of new mechanisms of resistance, both Bcr-Abl dependent and independent, requires always new therapeutic strategies to be improved, in order to guarantee increasingly higher survival rates. Therefore, this continuous challenge makes the topic discussed in the present review undoubtedly up-to-date. Interestingly, most of the studies so far reported were carried out with high-throughput screening, cellular and enzymatic assays, crystallography, nuclear magnetic resonance, mass spectrometry, and in silico predictions to determine selectivity spectrum, activity against mutations, and bioavailability of the new inhibitors in view of clinical translation. Noteworthy, these approaches can be applied to other targeted therapies facing specific resistance-conferring mutations in different models, e.g., T790M mutation of epidermal growth factor receptor (EGFR) against gefitinib [58] or several anaplastic lymphoma kinase (ALK) point mutations against crizotinib [59].

The increasing number of approved and experimental inhibitors is slowly approaching the therapeutic solution of single specific mutations of Bcr-Abl, which were also highly deadly until recent past, e.g., T315I; nevertheless, compound mutations are a further emerging hurdle in the clinic, with only little knowledge about their prognostic and therapeutic meaning, to date [60]. Indeed, they may need innovative approaches to be dealt with, such as combination therapies, reevaluating discarded TKIs (in light of new therapeutic demands) or molecules with wider spectrum of targets, e.g., danusertib. In light of this, strategies other than Bcr-Abl targeting may be successfully exploited against refractory diseases, as recently evidenced for IL-15-mimetic ALT-803, Hsp90-inhibitor ganetespib, HDAC-inhibitor panabinostat, Ras-antagonist rigosertib, β-catenin-antagonist PRI-724, and MELK-inhibitor OTS167, reviewed elsewhere [61]. Moreover, the hurdle of developing therapeutic monoclonal antibodies against CML may be overcome by evolving promising detection tools, as TPγ B9-2 towards protein tyrosine phosphatase receptor gamma (PTPRG) [62].

Another caveat that should be kept in mind is that probably “CML not always simply relies on t(9,22) Ph chromosome”: diverse translocations that activate Abl or other oncoproteins alternatively underlie very poor prognosis due to inefficacy of Bcr-Abl inhibitors [63, 64]. Conversely, other diseases characterized by hyperactivity of tyrosine kinases, such as PDGFRα in gastrointestinal stromal tumor (GIST) and hypereosinophilic syndrome or c-Kit in systemic mastocytosis, can benefit from imatinib and other inhibitors with wide selectivity spectrum [65]. Importantly, the mutational state of these kinases can drive the choice towards the most proper inhibitor: the commonly occurring imatinib-resistant PDGFRα D842V and c-Kit D816V mutants in GISTs and mastocytosis, respectively, are more strongly inhibited in vitro by dasatinib rather than imatinib (IC_50_ 62 vs 642 nM and 37 nM vs 3.8 μM, respectively—see Table 1 for these and other mutations), even though clinical benefits of dasatinib had been demonstrated only for the former [66]. However, the same therapy may work differently for diverse diseases, even if caused by similar genetic rearrangement: CML and Ph+ ALL are usually due to different Bcr-Abl forms, i.e., p210/p230 and p190, respectively [67]. Actually, it has been reported that p190 and p210 have at least partially independent signaling cascades that are mediated by differential protein-protein interactions, which may help explain the observed association of p190 with Ph+ ALL and p210/p230 with CML. Notably, the differential signaling networks of Bcr-Abl p210 and p190 kinases in leukemia cells have very recently been identified by functional proteomics, e.g., demonstrating a strong and preferential binding of AP2 complex, a major regulator of clathrin-mediated endocytosis, with p190, whereas Bcr-Abl is likely to be inhibited by p210-selective interactor Sts2 tyrosine phosphatase [68].

All that may also account for different therapeutic efficacy of specific inhibitors, as noticed while describing GNF modulators previously in the main text of this review.

Overall, the developmental process of Bcr-Abl inhibitors perfectly exemplifies an important concept of biomedical research: translational medicine, aimed at lading scientific breakthroughs from bench to bedside, drove the development of imatinib starting from the identification of target and pharmacophore; nonetheless, the more innovative concept of reverse translational medicine, aimed at turning “bedside” problems to “bench” ones, has been followed during the rational development of second- and third-generation TKIs to effectively override the progressive onset of resistances and adverse drug reactions in the clinic.

In the context of tumor therapy, those lessons learned from Bcr-Abl inhibitors serve both as a model to overcome the still open issues about CML and Ph+ ALL and as a proof of concept for rationally developing novel small molecules against specific tumor types.

Finally, imatinib history may serve as a milestone of the developmental process of any inhibitor, driving drug discovery towards future chemotherapy-free and target-oriented treatments.

## Abbreviations

ALK: Anaplastic lymphoma kinase
ALL: Acute lymphoblastic leukemia
ATP: Adenosine triphosphate
CML: Chronic myelogenous leukemia
DFG: Aspartate-phenylalanine-glycine
EGFR: Epidermal growth factor receptor
GIST: Gastrointestinal stromal tumor
H-b: Hydrogen bond
IC_50_: 50% inhibitory concentration
MMR: Major molecular response
PDGFR: Platelet-derived growth factor receptor
Ph: Philadelphia chromosome
PKC: Protein kinase C
TKI: Tyrosine kinase inhibitor
VdW: Van der Waals
WT: Wild-type
